# An Alginate Hybrid Sponge with High Thermal Stability: Its Flame Retardant Properties and Mechanism

**DOI:** 10.3390/polym11121973

**Published:** 2019-11-30

**Authors:** Yuhuan Jiang, Xuening Pang, Yujia Deng, Xiaolu Sun, Xihui Zhao, Peng Xu, Peiyuan Shao, Lei Zhang, Qun Li, Zichao Li

**Affiliations:** 1 College of Life Sciences, Institute of Advanced Cross-Field Science, Qingdao University, Qingdao 266071, China; 2018025210@qdu.edu.cn (Y.J.); 2018025216@qdu.edu.cn (X.P.); 2 College of Chemistry and Chemical Engineering, Qingdao University, Qingdao 266071, China; dengyujia@qdu.edu.cn (Y.D.); sunxiaolu6688@163.com (X.S.); zhaoxihui@qdu.edu.cn (X.Z.); X1627271005@163.com (P.X.); 15666681178@163.com (P.S.); 2017020832@qdu.edu.cn (L.Z.); 3 Shandong Collaborative Innovation Center of Marine Biobased Fibers and Ecological Textiles, Qingdao University, Qingdao 266071, China

**Keywords:** sponge, alginate, organic-inorganic hybrid materials, flame retardancy, thermal properties, mechanism

## Abstract

The worldwide applications of polyurethane (PU) and polystyrene (PS) sponge materials have been causing massive non-renewable resource consumption and huge loss of property and life due to its high flammability. Finding a biodegradable and regenerative sponge material with desirable thermal and flame retardant properties remains challenging to date. In this study, bio-based, renewable calcium alginate hybrid sponge materials (CAS) with high thermal stability and flame retardancy were fabricated through a simple, eco-friendly, in situ, chemical-foaming process at room temperature, followed by a facile and economical post-cross-linking method to obtain the organic-inorganic (CaCO_3_) hybrid materials. The microstructure of CAS showed desirable porous networks with a porosity rate of 70.3%, indicating that a great amount of raw materials can be saved to achieve remarkable cost control. The sponge materials reached a limiting oxygen index (LOI) of 39, which was greatly improved compared with common sponge. Moreover, with only 5% calcium carbonate content, the initial thermal degradation temperature of CAS was increased by 70 °C (from 150 to 220 °C), compared to that of calcium alginate, which met the requirements of high-temperature resistant and nonflammable materials. The thermal degradation mechanism of CAS was supposed based on the experimental data. The combined results suggest promising prospects for the application of CAS in a range of fields and the sponge materials provide an alternative for the commonly used PU and PS sponge materials.

## 1. Introduction

**Figure 1:**
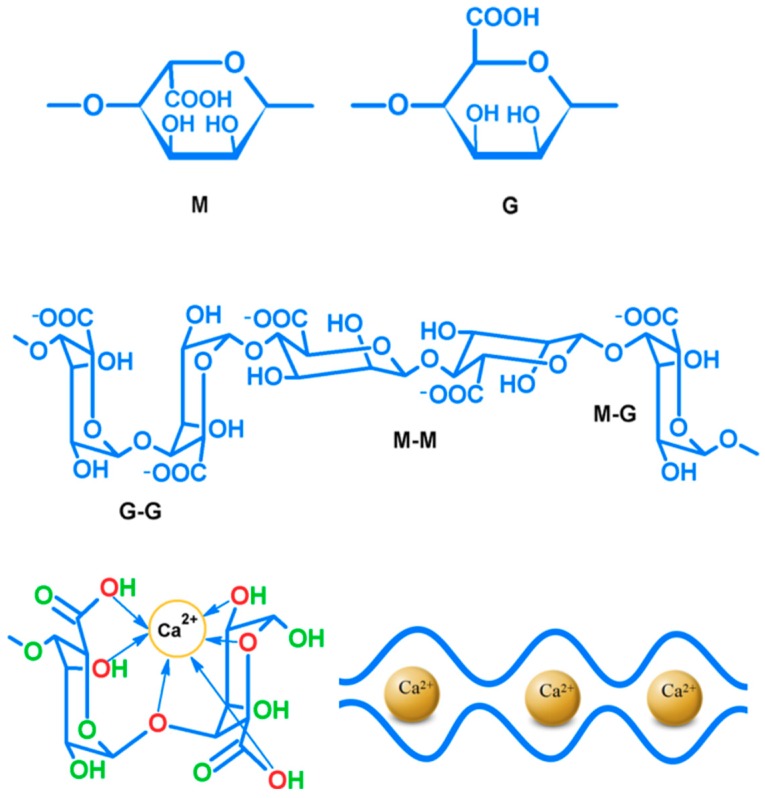
Structural diagram of alginate and calcium alginate.

The development of alginate has been arousing continuous interests as alginate is widely used in many industries, including food, textile printing, paper manufacture, wound dressings, and drug formulation, due to its low toxic, desirable mechanical and biocompatible properties [1,2]. As a natural linear polysaccharide, alginate is derived from seaweed, which is biodegradable and sustainable natural resources [3,4,5,6]. It is an anionic copolymer comprised of mannuronic acid (M block) and guluronic acid (G block) units arranged in an irregular structure of varying proportions of G-G, M-M and M-G blocks [7,8]. Chelation can be taken between multiple O atoms on the G block in alginate and Ca^2+^, forming a special kind of “egg box” structure (Figure 1) [9,10,11,12].

Gelation of alginate may happen by the interactions between carboxylate groups and divalent or trivalent metal ions in aqueous solution, forming strong, rigid, and ordered structures—the hydrogels [13,14]. Calcium alginate, barium alginate, copper alginate, and zinc alginate were reported to be inherent flame retardants, and the effects of these divalent metal ions on the flame retardant and thermal properties of alginate have been investigated [15,16,17,18,19]. Plenty of research has focused on water-insoluble calcium alginate, which can be easily produced via ion exchange reactions between Ca^2+^ and Na^+^ with favorable biodegradability and biocompatibility [20,21,22]. However, the extensive application, besides the food and pharmacy industries, of calcium alginate is limited by its poor thermal resistance and the high cost of sodium alginate as the raw material.

To date, polyurethane (PU) and polystyrene (PS) sponge materials have a wide range of applications in the processing of sofas, fitness mats, car seat cushions, packaging, shockproof materials, building insulation materials, etc. The annual output of PU sponge alone has reached about 20 million tons in recent years. However, these chemically synthesized materials not only consume a large amount of non-renewable petroleum resources, but also are flammable materials, causing enormous damage due fire annually. Therefore, it is of great importance to find an ideal alternative material with desirable thermal and flame retardant properties that is regenerative and biodegradable. A number of experimental methods for preparing sponge or foam materials with thermal insulation properties have been reported, however, the necessary freeze drying process below −20 °C in the preparation demands costly equipment and high energy consumption [23,24,25]. Moreover, layer-by-layer methods using green materials without affecting the structure and properties of basic materials have attracted plentiful attention recently [26,27,28]. Nonetheless, the complicated procedures of the manufacturing methods are difficult to scale up and thus are limited for industrial manufacturing.

In this study, in order to overcome the aforementioned shortcomings, we reported on a simple chemical foaming in situ method to prepare alginate hybrid sponge materials at room temperature. Related properties were conducted and analyzed by scanning electron microscopy (SEM), X-ray diffraction (XRD), Fourier transform infrared spectroscopy (FTIR), thermogravimetric analysis (TGA), cone calorimeter (CONE), and pyrolysis-gas chromatography-mass spectrometry analysis (Py-GC/MS). Furthermore, the proposed mechanisms of thermal degradation and flame retardancy based on the experimental data were investigated.

## 2. Experimental Section

### 2.1. Materials

Sodium alginate (food grade) was supplied by Guangfu Fine Chemical Research Institute (Tianjin, China). Calcium chloride anhydrous and sodium percarbonate were bought from Zhengcheng Chemicals Co., Ltd. (Tianjin, China). Manganese dioxide was purchased from Kermel Chemical Reagent Co., Ltd. (Tianjin, China). All the chemicals were analytical reagents and used without further purification.

### 2.2. Preparation of the Alginate Hybrid Sponge

**Scheme 1:**
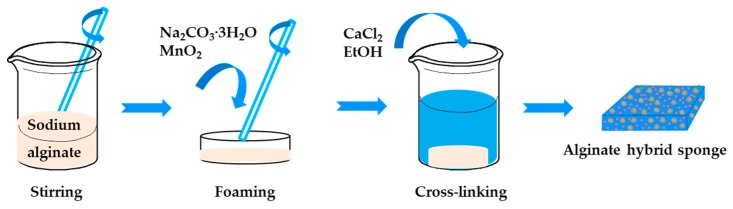
Preparation of the alginate hybrid sponge (CAS) via a chemical foaming and cross-linking method at room temperature.

The preparation procedure of the alginate hybrid sponge, including dissolution, foaming, cross-linking, and solidification processes, are shown schematically in Scheme 1. Briefly, sodium alginate was firstly dispersed in deionized water to form a 5 wt % sodium alginate solution with a vigorous stirrer at room temperature. Then, 50 mL solution was placed in a mold with the addition of 0.5 g of sodium percarbonate and trace amounts of manganese dioxide as the catalyst. After continuous stirring, the solution was foamed by a standing process for 45 min. The entirely foamed product was immersed in a 5% calcium chloride/ethanol solution at room temperature for 5 h to ensure that it was cross-linked completely and finally the alginate hybrid sponge (CAS) was obtained.

The preparation of calcium alginate (CA) was similar to that of CAS, except that the use of a foaming agent and catalyst was excluded in the process.

### 2.3. Characterization

A similar liquid displacement method reported by Guan et al. was employed to determine the porosity of CAS [29]. The mechanical properties, including breaking force (N) and elongation at break (%), were measured by a universal tensile machine (H5KL, Tinius Olsen Inc., Horsham, PA, USA) under tensile mode with a load cell of 250 N.

The morphology and microstructure of the samples and their thermal cracking residues at the different temperatures were characterized by scanning electron microscopy (SEM, JSM 6390LV, JEOL Inc., Tokyo, Japan).

X-ray diffraction (XRD) patterns of the samples were recorded on a powder X-ray diffractometer (D/Max-RB, Rigaku Inc., Tokyo, Japan), measured with Cu-Kα radiation (*λ* = 0.15418 nm), and scanned up to 80° in 2θ in a continuous mode.

Fourier transform infrared spectroscopy (FTIR, NICOLET iS10, Thermo Fisher Scientific Inc., Waltham, MA, USA) was used to determine the composition within the samples.

Thermogravimetric analysis (TGA) was performed on a thermogravimetric analyzer (TG 209, NETZSCH Geraetebau GmbH, Selb, Germany) at a heating rate of 10 °C min^−1^ and a final temperature of 900 °C under N_2_.

Pyrolysis-gas chromatography-mass spectrometry analysis (Py-GC/MS) was performed on a multifunctional pyrolyzer (EGA/PY-3030D, Multi-Shot Pyrolyzer, Frontier Laboratories, Ltd., Tokyo, Japan), connected to a GC system coupled with a MS detector (QP2010 Plus, Shimadzu Corporation, Kyoto, Japan). The pyrolysis temperature was set at 300 °C and 800 °C. Samples of 2 mg were pyrolyzed at a ramp rate of 20 °C min^−1^ with a hold time of 18 s. The products were separated in a DB-5MS (J&W Scientific Inc., Folsom, CA, USA) capillary column (length 30 m, inner diameter 0.25 mm, film thickness 0.25 μm), under a temperature of 40 °C with a hold time of 3 min, and heated to 300 °C at a ramp rate of 10 °C min^−1^ with a hold time of 20 min.

### 2.4. Measurements

The limiting oxygen index (LOI) values were measured on a JF−3 oxygen index meter (Jiangning, China) according to the standard method ISO 4589−1:1996. The specimens with dimension of 100 mm × 50 mm × 10 mm were used for the tests.

The combustion behavior was measured by an M1354 cone calorimeter device (Fire Testing Technology Ltd., East Grinstead, UK) according to ISO 5660, under an external heat flux of 50 kW·m^−2^. The size of all the tested samples was 100 mm × 100 mm × 10 mm.

Percentages of calcium content in the prepared samples and thermal cracking residues were determined by using the ethylenediaminetetraacetic acid (EDTA) method.

## 3. Results and Discussion

### 3.1. Characterization of the Sponge

**Figure 2:**
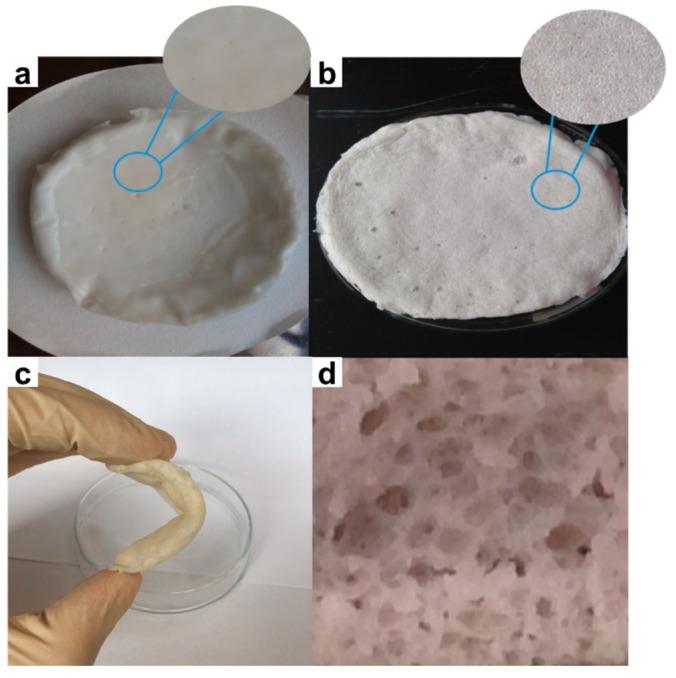
Photographs of (**a**) calcium alginate (CA), (**b**) alginate hybrid sponge (CAS), (**c**) elasticity of CAS, and (**d**) cross section of CAS. The insets in (**a**) and (**b**) are their magnified images, respectively.

As shown in Figure 2a, a rough surface without pores was observed in CA and the material was hard, with poor toughness, and could be easily broken by the external force. Comparatively, CAS showed a flat and porous surface and also the existence of a dense and homogenous porosity in the inner structure (Figure 2b,d). Moreover, CAS showed high elasticity, could be easily bent without being broken, and recovered when releasing the external force indicating its toughness (Figure 2c). Notably, the tested data of the mechanical and porosity properties for CAS, which are listed in Table 1, suggest a well fabricated sponge material.

**Figure 3:**
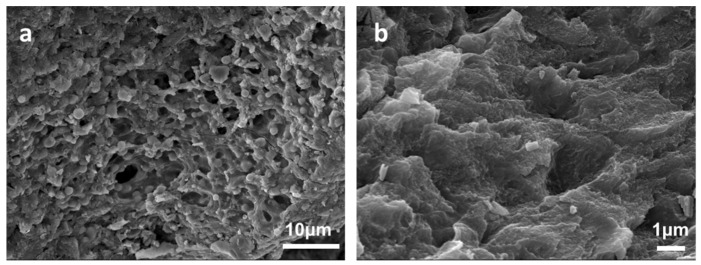
SEM images of (**a**) CAS and (**b**) CA.

To investigate the relationship between structure and properties, the micro morphologies of CAS and CA were determined by SEM. As shown in Figure 3, the surfaces of the two samples exhibited significant differences. For CAS, as displayed in Figure 3a, closed cellular structures with pore sizes of 1–2 μm were observed and were well distributed on the surface, indicating a successful foaming procedure designed for the sponge preparation, which was responsible for its high toughness and elasticity. Comparatively, as seen in Figure 3b, CA showed a typical polymer-like structure, with a moderate rough surface slightly paved by cracks, while no porosity existed [30,31].

**Figure 4:**
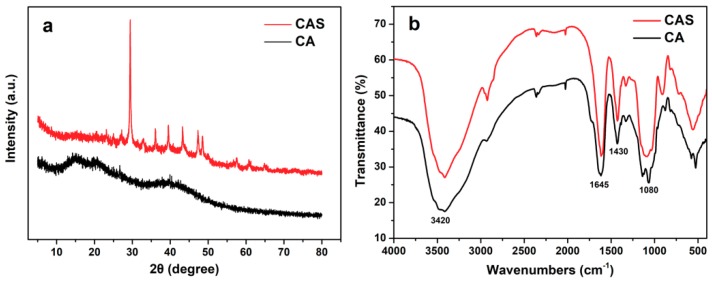
(**a**) XRD patterns and (**b)** FTIR spectra of CAS and CA.

X-ray diffractograms of the prepared samples are shown in Figure 4a. The XRD pattern for CA showed no crytalline compounds within the scanned 2θ range, and presented amorphous halos at 2θ of 13°, 22° and 40°, which were in accordance with the literature data [32]. A sharp peak at 29.5°, representing the calcite crystal of CaCO_3_, along with its characteristic peaks at 2θ of 36°, 39.5°, 43.5°, 48°, and 49°can be observed in the diffractogram of CAS, confirming the successful fabrication of calcium alginate/CaCO_3_ hybrid materials. As shown in Figure 4b, the FTIR spectrum of CA was similar to the previous report [31], while no noticable change in the characteristic absorption peak or intensity was observed when comparing the spectra of the two samples, indicating that the preparation of CAS by the catalytic foaming process was a physical change.

### 3.2. Thermal Stability

**Figure 5:**
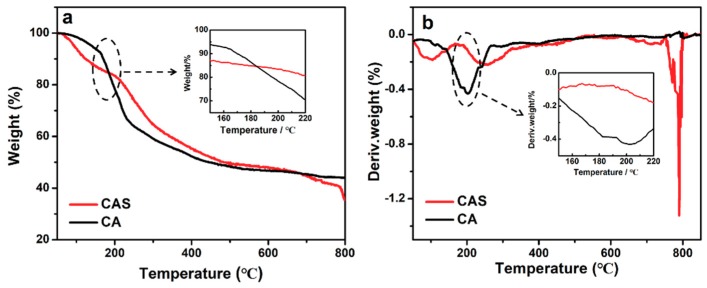
(**a**) Thermogravimetric analysis (TGA) weight loss and (**b**) derivative thermogravimetry (DTG) curves of CAS and CA.

TGA weight loss and derivative thermogravimetry (DTG) curves of CAS and CA are displayed in Figure 5. It can be seen from Figure 4a that, in the first stage, the weight loss rate for CA was about 6.1% at around 150 °C, while the one for CAS was about 12.6% at around 150 °C and 14.4% at 170 °C, with a weight loss peak at 120 °C, which can be attributed to its porous structure which absorbed more water, compared to CA [33]. In the second stage, CA began to lose weight at 150 °C and the degradation was nearly finished at around 280 °C. At 170–220 °C, the curve of CAS remained basically stable and once above 220 °C the second weight loss for CAS began. Subsequently, the third weight loss for CAS occurred at 760 °C and the thermal decomposition reaction of calcium carbonate was completed at 800 °C.

It can be inferred that the degradation mechanism of CAS was completely different from that of CA. On the one hand, more absorbed water in CAS was lost than in CA at the initial stage of heating. On the other hand, CA began to decompose at 150 °C while CAS began to at 220 °C, indicating its thermal stability was improved by 70 °C. The second degradation for CAS was completed after 350 °C, with a much lower weight loss peak, thus its heat resistance was remarkably enhanced. This improvement can make CAS less prone to cause fire and, even in the case of fire, CAS can delay fire spread due to its slow thermal degradation and production of less flammable gas, thus providing more valuable time for firefighting.

### 3.3. Combustion Behaviors

#### 3.3.1. LOI Analysis

LOI represents the minimum percentage of oxygen required for the combustion of samples in an oxygen-nitrogen atmosphere and is commonly used for the evaluation of the flammability and flame retardancy of polymeric materials in the same conditions [34,35]. A higher value of LOI reflects a better flame retardancy of the tested samples. The LOI values of CAS and CA were 39.7 and 35.8, respectively, while self-extinguishing were observed in the test for both of the samples, indicating the materials are nonflammable with low smoke generation. As the LOI of common PU sponge is only around 26, the results of CAS shows a great improvement of the flame retardant properties endowed by the successful hybrid of alginate and calcium carbonate.

#### 3.3.2. CONE

The combustion behaviors of the samples were studied using CONE, which is an effective performance-based and bench-scale method to evaluate flammability through simulating real-world fire conditions [36,37,38].

**Figure 6:**
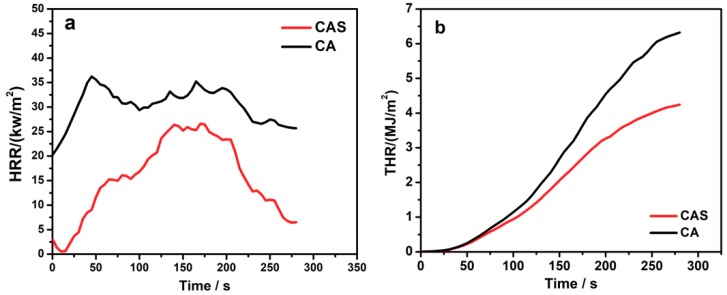
(**a**) Heat release rate (HRR) and (**b**) total heat release (THR) curves of alginate hybrid sponge (CAS) and calcium alginate (CA).

As shown in Figure 6a, overall, the heat release rate (HRR) value of CAS was lower than that of CA, which was in favor of slowing down the fire spread. In detail, CA showed an HRR peak at about 50 s, and continued for another 160 s. In contrast, the peak of CAS appeared at about 150 s and lasted for only 60 s. The 100 s delay may be caused by the enhancement of thermal stability. Meanwhile, the sharp decrease after 210 s for CAS can be associated with its endothermic property caused by thermal decomposition of CaCO_3_. It can be observed from Figure 6b that the total heat release (THR) increased linearly with time for both of the samples. However, the THR for CAS was lower than that of CA, comparatively. Moreover, the D-values between the two prepared samples increased with time, as listed in Table 2. In addition, the THR for CAS showed a much lower value of 4.00 kJ·m^−2^ at 250 s, compared with that for CA. The notable decreases in both HRR and THR for CAS indicate its much better flame retardancy than that of CA.

**Figure 7:**
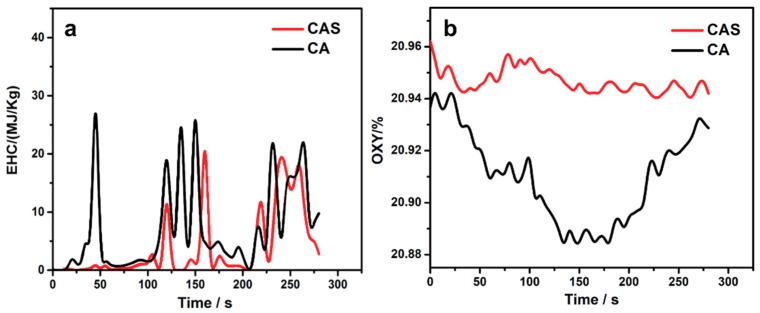
(**a**) Effective heat of combustion (EHC) and (**b**) oxygen concentration (OXY) curves of CAS and CA changing with time.

As seen in Figure 7a, the effective heat of combustion (EHC) peak of CAS, which was 27 MJ/kg, appeared at 50 s and it was continually exothermic from 100 to 150 s. In comparison, the first peak of CA appeared at 150 s, the height (12 MJ/kg) was less than half that of CAS, and it only exhibited another peak at 160 s. These results suggest different combustion mechanisms between the two samples. Furthermore, the oxygen concentration (OXY) curves for CAS and CA, displayed in Figure 7b, were completely different from each other, implying their different oxidation mechanisms. The OXY for CAS was hardly changed with a maximum decreasing peak value of only 0.06%, indicating that the oxidation reaction was not intense and mainly involved thermal decomposition weightlessness. In stark contrast, the OXY for CA exhibited a sharp decrease after 25 s and the maximum decreasing peak value appeared at 150 s lasting to about 200 s, suggesting that oxygen participated in the combustion reaction, resulting in a strong exothermic reaction, especially in the range of 130 to 180 s. This exothermic time horizon was in accordance with that of EHC curve between 100 and 150 s. The combined results support the different combustion and thermal degradation mechanisms for CAS and CA.

### 3.4. Characterization of Pyrolysis Residues

**Figure 8:**
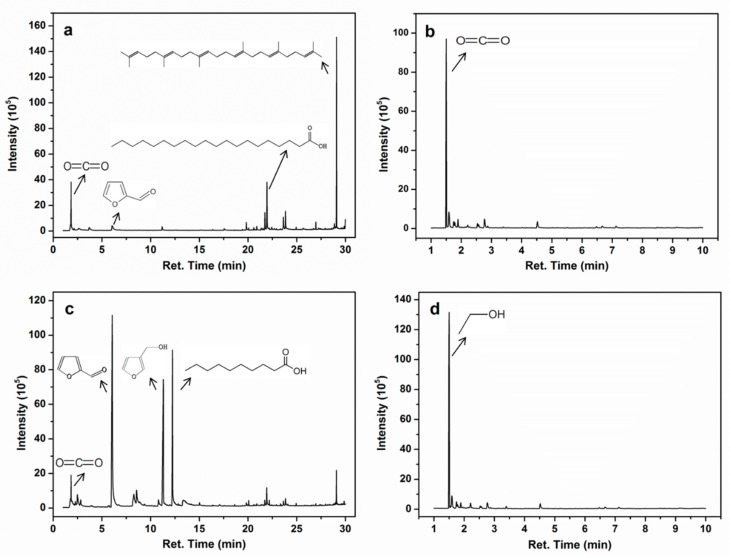
Pyrolysis-gas chromatography-mass spectrometry analysis (Py-GC/MS) spectra of CAS at (**a**) 300 °C and (**b**) 800 °C, and of CA at (**c**) 300 °C and (**d**) 800 °C.

The Py-GC/MS pyrograms at 300 and 800 °C for CAS and CA are compared in Figure 8. The main cracking products were identified by a mass spectral database and are listed in Table 3. It can be observed that the main pyrolysis products at 300 °C of CA were formic acid, furfural, and 3-furanmethanol, while for CAS, the main compounds such as acid, ester, and polyene hydrocarbon with long carbon chains were observed. Comparatively, the molecular weights of the main cracking products in CAS were greater than those of CA, suggesting a lower pyrolysis level, higher residual carbon rate, and better thermal stability of CAS under the same pyrolysis conditions. These results are in accordance with above mentioned TGA results, indicating the major contribution of CaCO_3_ in the improvement of heat resistance, pyrolysis rate, and pyrolysis level reduction in CAS.

At the higher pyrolysis temperature of 800 °C, few cracking products, mainly including ethanol and butadiene, were obtained from CA due to its high pyrolysis level with products of small molecular weight at 300 °C. Comparatively, more pyrolysis products with larger molecular weight, including cyclopentadienyl, alkadiene, and aromatic hydrocarbons, in CAS combustion were obtained due to the protection of CaCO_3_ at the same temperature. Noticeably, abundant CO_2_ was produced in the pyrolysis of CAS at 800 °C and this nonflammable gas can effectively dilute the concentration of flammable gas and thus inhibit fire spread. On the contrary, no CO_2_ was produced in the pyrolysis of CA at 800 °C, indicating that CaO was produced directly in its pyrolysis rather than CaCO_3_.

Collectively, it can be demonstrated that the CaCO_3_ generated by the catalytic foaming can remarkably improve the flame retardancy and inhibit the thermal degradation and pyrolysis of calcium alginate. Moreover, the pyrolysis mechanisms of the two samples are different due to their different composition and structure.

### 3.5. Thermal Degradation and Flame Retardant Mechanisms

#### 3.5.1. XRD and Surface Morphology Analysis of Thermal Degradation Products

It can be seen from the TG curve that 450 °C and 900 °C were the temperatures at which the calcium alginate and calcium carbonate were completely decomposed, respectively. In order to study the thermal degradation mechanism of CAS, XRD and SEM of the cracking products, heated up to the two temperatures with a hold time of 1 h, under an aerobic (employing muffle furnace) and an anaerobic (employing tube furnace, under Ar) atmosphere were performed. The residues were denoted as CAS 450, CAS 900, CA 450, and CA 900 for CAS and CA heated at the two set temperatures, respectively.

**Figure 9:**
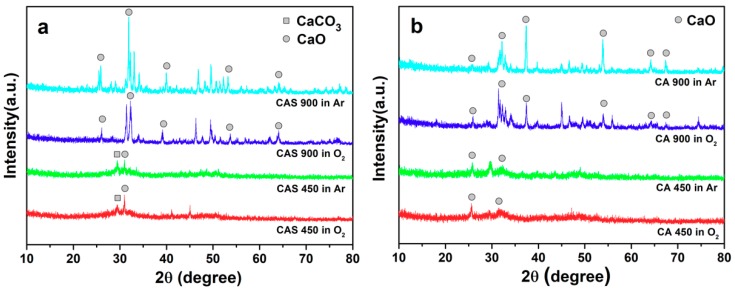
XRD patterns of the residues of (**a)** CAS and (**b**) CA. CAS 450, CAS heated up to 450 °C; CAS 900, CAS heated up to 900 °C; CA 450, CA heated up to 450 °C; CA 900, CA heated up to 900 °C.

The characteristic diffraction peaks of calcium oxide and calcium carbonate for CAS heated up to 450 °C with a hold time of 1 h are shown in Figure 9a. When the temperature reached 900 °C, the characteristic peak of calcium carbonate disappeared while that of calcium oxide was significantly enhanced. For CA, as seen in Figure 9b, only the characteristic peak of calcium oxide was observed despite the increase in temperature, indicating that calcium ions formed calcium oxide during the thermal cracking of the molecular chain in CA.

**Figure 10:**
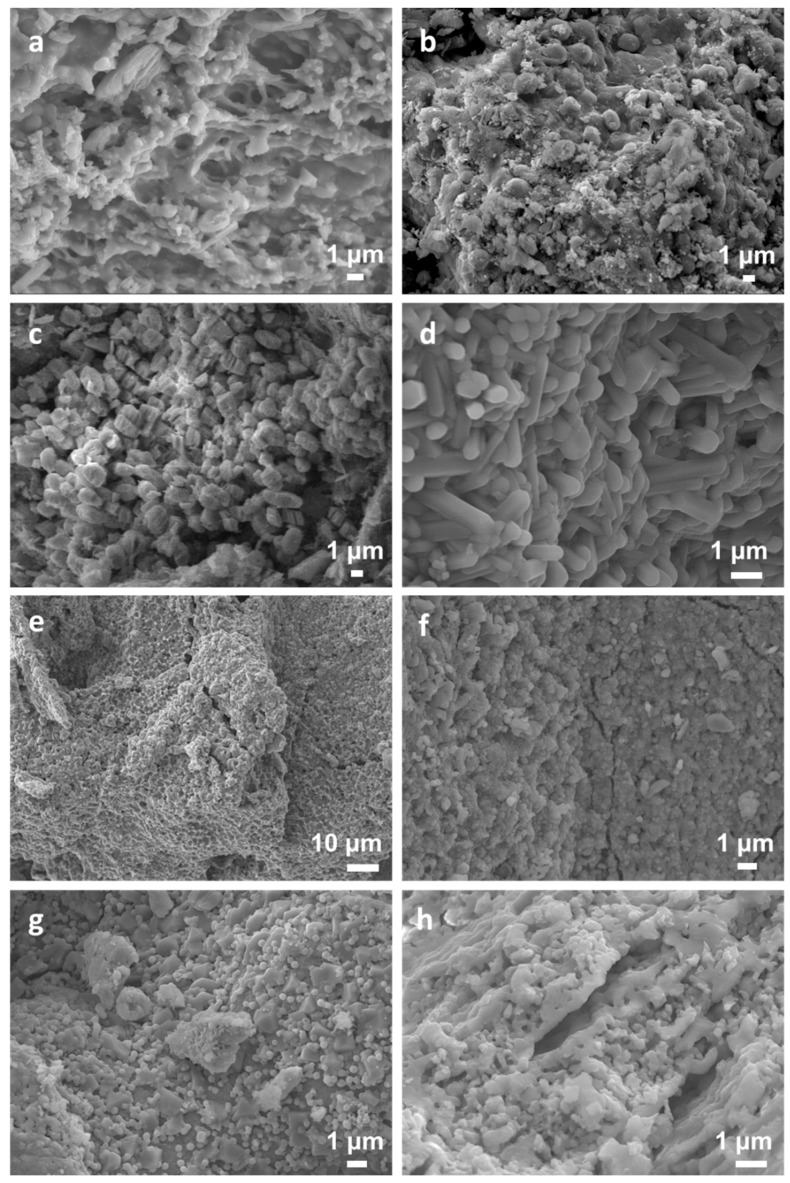
SEM images of the residues of the alginate hybrid sponge (CAS) and calcium alginate (CA). (**a**) CAS 450 under air, (**b**) CA450 under air, (**c**) CAS 450 under Ar, (**d**) CA 450 under Ar, (**e**) CAS 900 under air, (**f**) CA 900 under air, (**g**) CAS 900 under Ar, and (**h**) CA 900 under Ar.

It can be inferred from the XRD results that the aerobic and anaerobic atmospheres had little effect on the diffraction peaks for both of the samples. However, as observed from the SEM images displayed in Figure 10, the morphology of the decomposition products for CAS and CA under the two conditions was remarkably different. This may be because the char formed under anaerobic condition was beneficial to retain the pore structure while oxidation can destroy the pore structure and collapse the carbon skeleton.

As can be deduced from the above results, the degradation mechanism of CAS was completely different from that of CA. In considering thermal stability, CAS remained stable at 170–220 °C and the second weight loss peak appeared at 270 °C, with a much lower intensity than that of CA, while the degradation was completed at 350 °C. Thus, the thermal insulation property of CAS was significantly improved due to its unique porous structure.

#### 3.5.2. Supposed Mechanisms

**Scheme 2:**
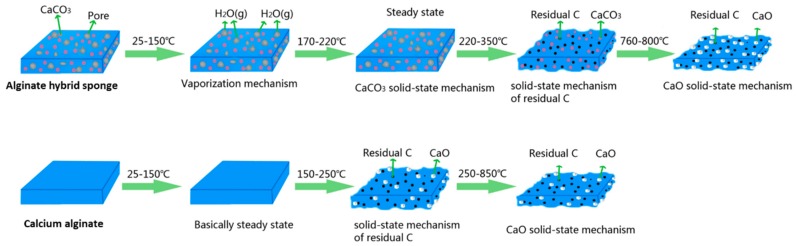
Schematic diagram of thermal degradation and flame retardant mechanisms for CAS and CA.

In combination with the aforementioned results, thermal degradation and flame retardant mechanisms of CAS and CA are illustrated in Scheme 2 and discussed below.

Considering the vapor phase flame retardant mechanism of water, for the first stage at 25–150 °C, CAS showed major losses in both absorption and crystallization of water. When the temperature was increased to 150 °C, the D-value between CAS and CA increased to 6.5%. Due to the release of water in gas form at the first stage, the greater weight loss for CAS can efficiently dilute the air and slow down the combustion rate.

In the thermal degradation process of calcium alginate and flame retardant reactivity of Ca^2+^ in solid phase, since sodium percarbonate was quantitatively added, according to the mass fraction (sodium carbonate/calcium ions = 1:1), the increment of calcium ion was 2%. The data for the variation in content of calcium ions, which was determined by the EDTA method during thermal degradation, are summarized in Table 4. It can be clearly seen that, with the increase in specific surface area of CAS, saturated absorption of calcium ions was increased by 0.8% and the calcium content in the residue of thermal degradation was increased by 2.8%. Of special note was the stability ranging from 170 to 220 °C for CAS in the second stage, indicating that calcium alginate was not yet degraded in this period. The thermal degradation for CA started at 150 °C and ended at 250 °C, while for CAS it was initiated at 220 °C and completed at 350 °C. Moreover, the TG curve of CAS was above that of CA, indicating that the thermal degradation rate decreased due to the existence of pores and calcium carbonate in CAS.

In the third stage, the thermal decomposition of calcium carbonate in CAS began at 760 °C, with a sharp peek at 790 °C, and ended at 800 °C. Theoretically, the thermodynamic decomposition temperature of calcium carbonate is 846 °C. The results indicated that the thermal decomposition temperature of calcium carbonate was reduced by 86 °C in the environment with carbon residues. Carbon dioxide and vapor played their major roles in the gas phase and the effects of surface-covering calcium carbonate decomposition and calcium oxide acted on the solid phase flame retardant mechanism. Moreover, the above synergetic effects of both gas and solid phases enhanced the thermal stability and flame retardant properties of CAS. With more water and calcium carbonate, CAS showed greater changes in thermal degradation behavior compared to that of CA, which are illustrated in Equations (1)–(3), where Q in Equation (3) is the heat of the reaction. The thermal degradation of calcium carbonate demanded the absorption of a large amount of heat, which was crucial to the improvement of heat resistance for CAS.

(1) $$\mathrm{CAS}\overset{\mathrm{reaction}\;\mathrm{temperature}>220\;{}^{\circ}C}{\underset{\mathrm{decarboxylation}}{\to }}\mathrm{alginate}\;\mathrm{decarboxylate}+\mathrm{CaO}+{\mathrm{CaCO}}_{3}+{\mathrm{CO}}_{2}$$

(2) $$\mathrm{CA}\overset{\mathrm{reaction}\;\mathrm{temperature}>220\;{}^{\circ}C}{\underset{\mathrm{decarboxylation}}{\to }}\mathrm{alginate}\;\mathrm{decarboxylate}+\mathrm{CaO}+{\mathrm{CO}}_{2}$$

(3) $${\mathrm{CaCO}}_{3}(\mathrm{foaming})\overset{\mathrm{reaction}\;\mathrm{temperature}\geq 760{}^{\circ}C}{\underset{\mathrm{decomposition}}{\to }}\mathrm{alginat}\;\mathrm{ecarbonization}+\mathrm{CaO}+{\mathrm{CO}}_{2}+Q$$

## 4. Conclusions

In summary, we have demonstrated a simple and environmentally-friendly in situ process to fabricate alginate hybrid sponge at room temperature by combining chemical foaming and post-cross-linking methods. Desirable porosity was achieved in the sponge materials and considerable costs can be cut for their production. The results of combustion behavior showed that the prepared sponges were materials with high thermal stability and low flammability, benefitting from the alginate hybridization with calcium carbonate. From the supposed mechanism, based on the experimental data, the generated carbon dioxide and vapor during the degradation of the sponge materials can reduce the density and temperature of the combustible gas in the gas phase, while the surface-covering calcium carbonate and calcium oxide, generated from the decomposition, can prevent the combustion reaction to achieve favorable flame retardancy in the solid phase. The as-made sponge materials exhibited excellent heat resistant and flame retardant properties while the preparation process was simple, eco-friendly, and economical. Hence, they have enormous potential in applications such as automotive interiors, fillings, children’s toys, and building insulation materials.

## Figures and Tables

**Table 1:** The mechanical and porosity properties of alginate hybrid sponge (CAS).

Sample	Thickness (mm)	Breaking Force (N)	Elongation at Break (%)	Porosity Rate (%)
CAS	7.0	17.2	29.6	70.3

**Table 2:** The total heat release (THR) of CAS and CA and their difference values (D-value) every 50 s.

Parameters	CAS	CA	D-Value
The THR at 100 s (kJ·m^−2^)	0.94	1.14	0.20
The THR at 150 s (kJ·m^−2^)	2.06	2.67	0.61
The THR at 200 s (kJ·m^−2^)	3.26	4.54	1.28
The THR at 250 s (kJ·m^−2^)	4.00	5.92	1.92

**Table 3:** Main cracking products of CAS and CA by pyrolysis-gas chromatography-mass spectrometry analysis (Py-GC/MS) at different temperatures.

Molecular Structure	T = 300 °C				T = 800 °C			
	CAS		CA		CAS		CA	
	Ret. Time (min)	Area (%)	Ret. Time (min)	Area (%)	Ret. Time (min)	Area (%)	Ret. Time (min)	Area (%)
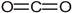	─	─	1.83	2.12	1.50	57.27	─	─
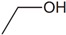	─	─	─	─	─	─	1.50	67.11
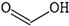	─	─	2.48	2.06	─	─	─	─
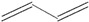	─	─	─	─	1.59	10.63	1.59	7.07
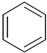	─	─	─	─	2.77	5.73	2.77	3.87
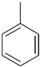	─	─	─	─	4.52	4.34	4.52	3.11
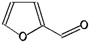	6.03	4.11	6.06	39.81	─	─	─	─
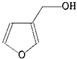	─	─	11.31	24.32	─	─	─	─
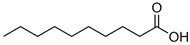	─	─	12.25	16.81	─	─	─	─
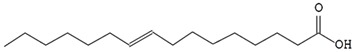	21.73	4.26	─	─	─	─	─	─
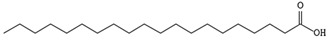	21.94	11.42	─	─	─	─	─	─
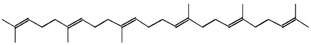	29.09	33.71	─	─	─	─	─	─
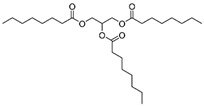	31.78	4.39						
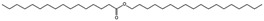	35.05	4.92						

**Table 4:** The calcium contents of CAS and CA.

Samples	Calcium Increment (%)	Residue (%)	Calcium in Residue (%)	Saturated Absorption of Calcium Increment (%)
CAS	2.0	27.0	13.6	0.8
CA	0	26.7	10.8	0
